# Functional Outcome in Athletes at Five Years of Arthroscopic Anterior Cruciate Ligament Reconstruction

**DOI:** 10.5402/2011/570329

**Published:** 2011-07-03

**Authors:** Ashish Devgan, N. K. Magu, R. C. Siwach, Rajesh Rohilla, S. S. Sangwan

**Affiliations:** Department of Orthopaedics, Pt. B. D. Sharma University of Health Sciences, Rohtak, Medical Campus, Haryana, India

## Abstract

*Introduction*. The purpose of this study was to analyze the functional outcome in competitive level athletes at 5 years after ACL reconstruction with regard to return to sports and the factors or reasons in those who either stopped sports or showed a fall in their sporting levels. *Methods*. 48 competitive athletes who had undergone arthroscopic assisted ACL reconstruction with a minimum follow up of at least 5 years were successfully recalled and were analyzed. *Results*. 22 patients had returned to the preinjury levels of sports and 18 showed a decrease in their sporting levels. Of the 18 patients, 12 referred to fear of reinjuring the same or contra-lateral knee as the prime reason for the same while 6 patients reported persisting knee pain and instability as reasons for a fall in their sporting abilities. The difference in the scores of these groups was statistically significant. 8 patients out of the 48 had left sports completely due to reasons other than sports, even though they had good knee outcome scores. *Conclusion*. Fear of reinjury and psychosocial issues that are relevant to the social milieu of the athlete are very important and affect the overall results of the surgery with respect to return to sports.

## 1. Introduction

The sporting career of an athlete depends not only on how soon he can return back to his sporting activity but also on the level of return to sports, with the least long-term complications. Rupture of anterior cruciate ligament (ACL) results in a mechanically unstable joint, resulting in difficulty in athletic performance [1], increased risk of subsequent meniscal injury [2], and increased risk of early degenerative joint disease [3]. ACL reconstruction is recommended in athletes to help restore knee stability for return to pivoting sports. Many different techniques using a variety of grafts with varying fixation techniques have continued to evolve to restore the stability to an ACL-deficient knee. Numerous papers and meta-analyses have shown similar results by different graft materials using multiple graft fixation techniques [4–9].

However, the results on return to sports after ACL reconstruction have varied [10–12]. Although short-term evaluation is critical for assessment with regard to return to sports, an assessment 5 to 6 years after surgery is essential to determine the medium to long-term effect of surgery on maintaining knee joint stability, range of motion, restoring patient satisfaction while on field, returning to stressful pivoting sports, and development of complications if any. The ability to return to sports after ACL reconstruction is governed not only by postoperative knee function but also by various other factors like social reasons, psychological impediments like fear of reinjury, and even monetary factors especially in sports persons of developing countries. 

There is a dearth of Western literature regarding return to sports after surgery that is relevant to Indian context making it difficult to counsel our patients regarding their eventual return to sports. To the best of our knowledge there is no study that evaluates the mid- to long-term results regarding return to sports after ACL reconstruction in Indian sports persons. 

The purpose of this study was to analyze the functional outcome in competitive level sports persons at 5 years after ACL reconstruction. Our hypothesis was that reliable and sustainable results could be achieved over time using the arthroscopic technique of ACL reconstruction. Additional goals were to assess function in the ACL reconstructed knee, return to sports and level of sporting activity, patient satisfaction, identification of complications if any, and the factors or reasons in those who either stopped sports or showed a fall in their sporting levels. 

## 2. Patients and Methods

Between 2002 and 2005, records of 96 patients who underwent arthroscopic ACL reconstruction by the single surgeon (first author) were procured. We proceeded to recall 68 of them who were sports persons. Patients with concomitant meniscal and chondral lesions were included whilst excluding those with multiligament injuries. 62 persons could be contacted out of whom 48 persons agreed to come for followup examination and interview. The mean age of our patients was 23.6 years (range 20.4 to 28.7 years). There were 44 males and 4 females. All were involved in competitive level sports at district and state level including 6 who were national level athletes. The sports played were Wrestling (32 patients), Kabaddi (8), Athletics (6), and Cricket (2). All had symptomatic and repeated episodes of instability despite conservative treatment and had wished to return to competitive sports that involved pivoting, cutting, and side stepping actions before proceeding to surgical procedure. 

All patients of our cohort underwent arthroscopic ACL reconstruction using single incision transtibial technique by a single surgeon. 26 underwent ACL reconstruction using bone patellar tendon and 22 using hamstring graft. Standard titanium interference screws were employed for fixation of patellar tendon graft with additional cortical screw post on the tibial side. For the hamstring grafts Endobutton (Smith & Nephew, Mass, USA) was used for fixation on the femoral side whilst using a biodegradable screw with tendon staple on the tibial side. Meniscal tears were managed by simultaneous partial or subltotal meniscectomy as required. Chondral lesions were treated by debridement and microfracture technique. 20 patients who had meniscal or chondral lesions or both were subgrouped and were compared with those patients who only underwent arthroscopic ACL reconstruction 

The post operative program was standardized in all cases that involved quadriceps and hamstring isometric setting exercises, progressing to closed chain exercises and range of motion physiotherapy with the aim of regaining full range of motion by 6 weeks. Partial weight bearing was allowed at 3-4 weeks and light running on even ground, cycling, semi squats, and step exercises after 6 weeks. At 16 weeks, in addition to the strengthening exercises, sports-specific physiotherapy was instituted. Return to sports involving pivoting, cutting, or side stepping was permitted at 6 months after surgery if the patient had close to full range of motion and muscle strength.

The patients were clinically examined and completed the Subjective International Knee Documentation Committee (IKDC) questionnaires, the Lysholm Knee Form, and the Tegner Activity Scale (TAS). Clinical examination was performed according to the Objective IKDC evaluation form.

The IKDC rating scale [13] has both subjective questionnaire and an objective evaluation form. The IKDC subjective score is a questionnaire with different subjective factors such as symptoms, sports activities, and ability to function. The objective IKDC grading has 7 parameters related to the knee, reflecting both impairments and disability. The worst grading for the first three key parameters, that is, presence of effusion, knee range of motion, and ligament stability, determines the final IKDC grade. There are 4 grades—A, B, C, and D implying, respectively, normal, nearly normal, abnormal, and severely abnormal.

The Lysholm Knee Score [14] quantitates knee function, symptoms, and disability in a scale of 1 to 100 points, with 100 implying the best results and 1 the worst results. 

The Tegner Activity Scale [14] depicts the level of sporting activity and allows us to compare and document the preinjury activity level with the present activity level*.*

All the patients in our cohort had preinjury TAS level of 7 or more, which indicates that they were involved in competitive sports. At the time of review, they were asked whether they were still playing sports and whether they had returned to their preinjury levels of sporting activity. Return to sports was defined as returning to the same preinjury type and level of sports. Those patients who either stopped sports or showed a decrease in level of participation were asked to tell the reasons for the same. The group of patients who returned to the same level of sports was compared with the group of patients who either stopped sports completely or decreased their level of sporting activity. 

Statistical analyses using Chi-square with Yates' correction and one way analysis of variance (ANOVA) test for independent samples were performed to compare results in patient groups to determine if the reasons for not returning to sports had any significant correlation to the documented objective and subjective scales. 

## 3. Results

At 5-year followup, the mean Lysholm score was 86.4 (SD = 8.8). The mean subjective IKDC score was 82.8 (SD = 14.8). 84.6% of patients had normal or nearly normal objective IKDC grade (A or B), while the remaining 15.4% had IKDC grade C (abnormal). The median preinjury Tegner Scale was 8 (SD = 1.1), and the median 5 years after ACL reconstruction Tegner Scale was 7 (SD = 1.8).

8 patients out of the 48 that were reviewed at 5 years had left sports completely due to reasons other than sports. These included social reasons like marriage, getting into police and military services, and monetary reasons. Out of the remaining 40, 22 patients had returned to the preinjury levels of sports and 18 showed a decrease in their sporting levels. Of the 18 patients when asked for reasons for fall in sporting levels, 12 refered to fear of reinjuring the same or contra-lateral knee as the prime reason for the same. 6 patients refered to persisting knee pain, instability, annoying clicks, and numbness around the joint as reasons for a fall in their sporting abilities.  Table 1 shows the groups of patients according to their return to sports.

Table 2 shows the results of various scores in the patient subgroups according to Table 1. We found that at 5-year followup, the subgroup of patients that had returned to preinjury level of sporting activity (45.8%) had the best scores—IKDC grade A and B 95.4%, Lysholm 89.4, and subjective IKDC 87.6. This was in contrast to patients who showed a fall in their sporting levels because of painful and unstable knee (12.5%). At 5-year followup, they had the lowest scores—Lysholm 74.3, subjective IKDC 64.6, and objective IKDC grade A and B 33.3%. Those patients who decreased their sporting levels due to fear of re-injuring their knee (same or contra-lateral) (25%) showed that they had intermediate scores—Lysholm 82.3, subjective IKDC 76.7, and IKDC Grade A and B 75%. 

By statistical analyses, the difference in the outcome scores in the aforesaid three categories of patients was found to be statistically significant—objective IKDC, subjective IKDC, and Lysholm scale (*P* <  0.05) (Table 2).

There was another group of patients who stopped competitive sports completely (16.6%). When we see their scores we find that Lysholm was 88.9, subjective IKDC 86.4, and IKDC A and B 100%. They cited social reasons like marriage, monetary factors, and getting into police and military services as the main reasons for not continuing with sports despite having scores that were comparable with patients who returned to their preinjury levels of sporting levels (Table 2). 

## 4. Discussion

By this study we have reviewed the functional results at 5 years after arthroscopic ACL reconstruction in a cohort of competitive level sports persons. The results that were quantitated by Lysholm, subjective and objective IKDC, and Tegner Activity Scale were comparable to those in previously published studies [15, 16]. In our study, subjective assessment with particular attention to return to sports and at what level was given more attention than objective findings, type of graft used, fixation method used, instrumented testing of joint stability, and investigations like roentogram. 

Noyes et al. [1] proposed the rule of thirds for chronic ACL injury managed conservatively with rehabilitation and physiotherapy. They stated that one third of their patients resumed their previous recreational activities without reconstruction, one third managed by modifying their activity level and one third required reconstruction because of recurrent giving-way episodes even in day-to-day activities. Myklebust et al. [17] showed in their followup of competitive hand ball players that 91% of players treated without reconstruction could return to their preinjury activity level compared to 58% in the reconstructed group. Satku et al. [18], however, found that at 6 years after ACL injury, only 46% of their patients treated without reconstruction could return to preinjury sports. Kostogiannis et al. [19] indicated that many in their cohort who returned to sports at the same Tegner level without reconstruction avoided contact sports as advised by the rehabilitation team. These kind of conflicting results in the literature create confusion in the mind of the attending surgeon who is counseling the injured sports person for ACL reconstruction. However, the consensus rests on the suggestion that an athlete who wishes to return to his preinjury level should undergo reconstruction, especially competitive athletes or individuals engaging in pivoting sports [11, 12].

The literature is also full of a variety of grafts and fixation devices that are employed for arthroscopic ACL reconstruction [4–9]. However most of them show similar results regarding stability, patient function, and final outcome. The median TAS before injury in our patients was 8 and at five-year review it was 7. The mean TAS before injury was 7.72 ± 1.1 (range 6–9). The mean TAS at review was 6.92 ± 1.38 (range 3–8). This is comparable to studies by Ejerhed et al. [20] −6 and Charlton et al. [21] −5.7. The mean subjective IKDC score was 82.8. This is comparable to that of 84.3 of Matsumoto et al. [22] and Charlton et al. [21] −83. 84.6% of patients had normal or nearly normal objective IKDC grade (A or B). Studies by Jaeger et al. [23] (−89%), and Charlton et al. [21] (−91%) show slightly better results in the objective score. The mean Lysholm score was 86.4 as compared to 91.1-Jaeger et al. [23], and 91-Charlton et al. [21]. 

Return to sports is one of the most important outcome measures of a successful ACL reconstructive procedure. In our study, 22 (45.8%) of the patients who underwent subjective and objective analyses at 5 years after their ACL reconstruction had returned to their preinjury levels of sporting activities. In the literature the data for return to sports shows a wide variation—51% (Maletis et al. [4]), 53% (Kvist et al. [10]), 65% (Gobbi and Francisco [11]), 71.4% (Smith et al. [12]), 92% (Nakayama et al. [24]), and 100% (Fabbriciani et al. [25]). The literature also shows that competitive level athletes are more likely to return to the same level of sports after ACL reconstruction as compared to recreational level athletes. This may be one of the factors that account for a wide variation of percentage of return to sports as depicted in the literature. Fabbriciani et al. [25] report a 100% return of their cohort of 18 competitive level rugby players to the same level of sports after ACL reconstruction at 6-month and at 2-year followup. The motivation to return to sports is very high especially in competitive sports persons after surgery. Smith et al. [12] reported that 81% of their patients who were competitive athletes returned to sports within 1 year of surgery. However, at mean followup at 43 months after surgery, this dropped to 71% of their initial cohort. Another interesting point was that 21.8% were still in sports despite major functional impairment in the operated knee. This study highlights the fact that a very high motivational factor may be the reason for a high return ration in competitive athletes. Also there is a significant fall in percentage when reviewed at 1 and at approximately 3 years after surgery. Thus assessment regarding return to sports should not only be a shortterm one but should also look at mid- to long-term results vis-visa return to sports. Our study reviews the results at 5-years after reconstructive surgery in competitive athletes. 

However, in our study of competitive athletes the return to sports was only 45.8%, due to reasons other than sports that include social reasons, monetary reasons, and fear of reinjury to the same or contra-lateral knee. In our study, if we exclude the 8 (16.6%) cases who had a stable symptom-free knee but had left sports due to social and other reasons, our results show that 55% of cases returned to preinjury levels of sports. This appears a low rate of return as compared to the literature. But all these are Western literature, where there are a Dedicated team of sports-specific physiotherapists, a sports-specific psychologist to counsel the patients, and ample funding from government, and private sources to support the surgical costs, physiotherapy and rehabilitation of the injured sports person. Fear of reinjuring their knees and going through the surgery again, a prolonged period of physiotherapy, and remaining off the competitive field of sports proves to be a detrimental factor in the minds of our patients. This factor was found to be a major factor that led to fall in sporting levels in 12 of the 18 patients who showed a fall in their sporting levels at 5-year followup after arthroscopic ACL reconstruction. The same has been observed by Kvist et al. [10] and Lee et al. [26]. Asano et al. [27] reported that 66.1% of their patients experienced fear of re-injury at 9.3 months. Rathinam et al. [28] reported that 72% of their patients who did not return to their preinjury levels of sporting activities feared instability. A striking point was that the majority (70%) of them had no objective knee instability. In our study, fear of re-injuring the same or contralateral knee was a major factor in 12 of the 18 cases who showed a fall in sporting activities at 5-year review. It has been observed also in our study where the results of various scores in the sub group of patients who had fear of re-injury were not poor but were intermediate (Table 2) that is they were better than those who had a painful and unstable knee but worse than those who had returned to their preinjury sporting levels. 

Our study highlights an area that is often forgotten in the rehabilitation and evaluation after ACL injury or reconstruction. No attempts are made to find the reasons for fear of disability to return to sports. Plausible factors that have not been evaluated are, for example, impaired knee proprioception and neuromuscular control possibly resulting in both decreased performance and increased fear of re-injury. The number of injured knee structures, objective knee stability, time between injury and ACL reconstruction, and follow-up time are important factors that may influence performance [29]. The long rehabilitation time and difficulties to regain a position in the sports team may affect motivation and cease the athlete's competitive career in favour of social and family life [30]. Further prospective research combining assessments of psychological variables and functional tests is warranted in order to fully elucidate why patients return or not to their preinjury level and to fully establish the reasons.

The Tampa Scale of Kinesiophobia (TSK) has been used by Kvist et al. [10] to quantify the fear of re-injury due to physical activity. Their study reports a 53% return to preinjury level of sports after 3 to 4 years of ACL reconstruction. A high score on TSK scale implying a greater fear of re-injury and pain correlated with patients who did not return to preinjury level of sports. 

The other group of 6 out of 18 patients who showed a fall in sporting levels pointed a painful and unstable knee as reasons for the same. This has been reflected very well in their outcome scores (Table 2) which show poor subjective and objective scores. When we look at the three groups—those who returned to preinjury level, those who showed a fall due to a painful, unstable knee, and those who showed a fall in levels due to fear of re-injury—we find that the difference in the scores of the three groups was significant statistically. Possible factors that have been suggested for this are impaired knee proprioception and neuromuscular control leading to decreased performance and increased fear of re-injury [10]. 

Our study highlights that the psychosocial issues that are relevant to the social milieu of the athlete are very important and affect the overall results of the surgery with respect to return to sports. Socioeconomic pressures are cited to be a major factor especially in a developing country. Moreover, we found that there was a psychological fear in the mind of the athlete that his knee is weak and he can reinjure it more easily than the normal knee. The whole thing makes him afraid of rerupturing the graft as well as injuring the ligament in the contralateral knee as well. 8 of our patients left sports completely although on assessment their knees had good outcome scores (Table 2). A thorough and in-depth counseling by the surgeon at the time of index surgery besides social and family support mechanisms including regular sport-specific physiotherapy and psycho therapy session prove to be of great help in this regard. 

Our study has limitations in the form of a short sample size and a high drop-out rate of followup at 5 years. A young, active population that undergoes this surgery has high relocation rates due to study and employment reasons. Long-term studies related to orthopedic sports medicine well document this problem of loss to follow-up. A national or regional level ACL registry to follow up cases after surgery is called for in the current scenario. Despite the limitations, our study should prove useful to orthopedicians who operate and treat sports persons as they counsel them for surgery regarding the likelihood of eventual return to sports. 

## Figures and Tables

**Table 1:** Distribution of patients according to return to sports.

Total number of patients reviewed at 5-year followup	48

Number of patients who left sports completely due to other reasons	8

Number of patients who returned to the same level of sports	22

Number of patients who showed a fall in sporting levels	18

**Table 2:** Functional and clinical outcome scores.

Group (number of patients)	Lysholm score	IKDC Subjective	IKDC objective				Tegner scale
			A	B	C	D	
Return to sports at preinjury levels (22)	89.4	87.6	12	9	1	—	7

Fall in sporting activity due to fear of re-injury (12)	82.3	76.7	—	9	3	—	6

Fall in sporting activity due to Painful/unstable knee (6)	74.3	64.6	—	2	4	—	3

*P* value	<0.0001	<0.0001	0.004				<0.0001

Left sports completely due to social/other reasons (8)	88.9	86.4	2	6	—	—	Left sports

*P* value shows statistical difference between three groups, that is, those who returned to sports at preinjury levels (*n* = 22), those who had fall in sporting activity due to fear of re-injury (*n* = 12), and those who had fall in sporting activity due to painful/unstable knee (*n* = 6).
